# Preparation, Characterization and Application of Active Food Packaging Films Based on Sodium Alginate and Twelve Varieties of Mandarin Peel Powder

**DOI:** 10.3390/foods13081174

**Published:** 2024-04-12

**Authors:** Dawei Yun, Jun Liu

**Affiliations:** College of Food Science and Engineering, Yangzhou University, Yangzhou 225127, China; daweiyun2021@126.com

**Keywords:** active packaging, mandarin peel power, mandarin variety, sodium alginate, edible oil packaging

## Abstract

The industrial processing of mandarin fruits yields a large amount of peel waste, resulting in economic losses and environmental pollution. The peels of mandarin fruits are a good source of biomass and active substances that can be used to produce food packaging systems. In this study, active food packaging films were prepared based on sodium alginate and twelve varieties of mandarin peel powder. The structures, properties, and corn oil packaging performance of the films were compared. Results showed that the twelve varieties of mandarin peel powder differed in pectin, lipid, protein, crude fiber, and total phenol contents. The prepared films all exhibited a yellow color, 117.73–152.45 μm thickness, 16.39–23.62% moisture content, 26.03–90.75° water contact angle, 5.38–8.31 × 10^−11^ g m^−1^ s^−1^ Pa^−1^ water vapor permeability, 5.26–12.91 × 10^−20^ m^2^ s^−1^ Pa^−1^ oxygen permeability, 4.87–7.90 MPa tensile strength, and 13.37–24.62% elongation at break. Notably, the films containing mandarin peel powder with high pectin and lipid contents showed high moisture/oxygen barrier ability and mechanical properties. The films containing mandarin peel powder with high total phenol content exhibited high antioxidant- and antimicrobial-releasing abilities and good performance in delaying corn oil oxidation. Overall, the results suggested that the films have good application potential in active food packaging.

## 1. Introduction

Traditional food packaging materials play passive protective roles in food storage, acting as barriers to environmental impacts (e.g., dust, moisture, gas, and contamination) [1]. Nowadays, scientists have shifted their focuses from traditional packaging to active packaging, where the packaging materials can provide active protective functions for food [2]. There are two ways to actively protect food through active packaging: (1) absorbing or scavenging undesired gas, odors, and moisture from food or the packaging environment; and (2) emitting antimicrobial and antioxidant agents into food or the headspace of the package [3]. Accordingly, many active packaging techniques including oxygen-scavenging, ethylene-absorbing, moisture-controlling, antioxidant-releasing, and antimicrobial-releasing systems have been developed [4]. These active packaging systems show good potential in ensuring food quality and extending food shelf life.

In the active packaging sector, antioxidant-releasing and antimicrobial-releasing systems have received the greatest level of attention because oxidation and microbial contamination are two primary causes of food deterioration [5]. Antioxidant-releasing and antimicrobial-releasing systems are normally prepared by integrating antioxidant and antimicrobial substances in biodegradable polymer-based solid supports [6]. These antioxidant and antimicrobial substances can be released from packaging systems and enter food or the headspace of the package, thereby retarding food oxidation and inhibiting microbial contamination in direct or indirect manners [7]. In general, natural antioxidant and antimicrobial substances (e.g., essential oils and polyphenols) are more favored in active packaging, as compared with synthetic ones [8,9]. However, the use of natural antimicrobial and antioxidant substances normally involves tedious steps, such as extraction, isolation, and stabilization, which greatly increase the cost of active packaging production [10]. Therefore, it needs to seek alternative methods to prepare antioxidant-releasing and antimicrobial-releasing systems by avoiding the extraction and isolation of active substances.

In recent years, agricultural wastes (especially the processing residues of vegetables and fruits) have been considered as suitable raw materials for the production of antioxidant-releasing and antimicrobial-releasing systems [11]. The use of agricultural wastes in packaging systems has several advantages: (1) Agricultural wastes are a good source of biomass (e.g., carbohydrate, lipid, and protein) and active substances (e.g., essential oils and polyphenols) that can individually perform as the matrix and functional agents of packaging systems; (2) Agricultural wastes can be simply pretreated (e.g., dried and milled) and then be directly used to produce packaging systems, which remarkably reduces production cost; (3) The fabrication of packaging systems with agricultural wastes can alleviate the environmental burden of waste disposal [12,13]. Thus, the use of agricultural waste is a feasible and sustainable way to produce value-added active packaging systems.

Citrus fruits are commonly cultivated and consumed crops worldwide [14]. The industrial processing of citrus fruits yields a large amount of peel waste, resulting in economic losses and environmental pollution. Notably, citrus peels are rich in biomass (e.g., pectin, protein, lipid, and crude fiber) and active substances (e.g., polyphenols) [15]. To date, many studies have been conducted on the preparation of active packaging films using citrus peels [16,17,18,19,20,21,22]. In our previous study, active packaging films were produced based on sodium alginate and the peel powder of four common citrus fruits (i.e., mandarin, pomelo, lemon and orange). Results showed the films containing mandarin peel powder had the highest antioxidant- and antimicrobial-releasing abilities [23]. It is worth noting that dozens of mandarin varieties are cultivated in China. The variety of mandarin greatly affects the proximate composition of mandarin peel [14]. In view of this, active packaging films produced from sodium alginate and different varieties of mandarin peel powder might have different structures and properties.

In this study, active food packaging films were produced from sodium alginate and twelve varieties of mandarin peel powder. The aims of this study were: (1) to compare the structures and properties of the films; (2) to analyze the correlations between the component content of mandarin peel powders and the structures and properties of the films; (3) to verify the packaging performance of the films on corn oil.

## 2. Materials and Methods

### 2.1. Materials and Chemicals

Sodium alginate (average molecular weight of 710 kDa), glycerol, 6-hydroxy-2,5,7,8-tetramethylchroman-2-carboxylic acid (Trolox), gallic acid, and 2,2-diphenyl-1-pyrrolhydrazyl (DPPH) were purchased from Macklin Corp. (Shanghai, China). Twelve varieties of fresh mandarin fruits including Bendizao (BDZ, *Citrus succosa* Hort), Chunjian (CJ, *Citrus reticulate* Blanco ‘Chunjian’), Dahongpao (DHP, *Citrus reticulata* ‘Dahongpao’), Gonggan (GG, *Citrus reticulata* Blanco var. gonggan), Jiaogan (JG, *Citrus reticulata* Blanco ‘Tankan’), Nanfeng (NF, *Citrus reticulata* Blanco cv. Kinokuni), Ougan (OG, *Citrus reticulate* cv. *Suavissima*), Ponkan (PK, *Citrus poonensis* Hort. ex Tanaka), Shatangju (STG, *Citrus tachibana* Blanco), Shimen (SM, *Citrus unshiu* Marc.), Wogan (WG, *Citrus reticulata* cv. Orah), and Yichang (YC, *Citrus ichangensis Swingle*) were collected from different production regions of mandarin in China. Corn oil was bought from Yonghui Supermarket (Yangzhou, China).

### 2.2. Preparation and Compositional Analysis of Mandarin Peel Powder

**Figure 1:**
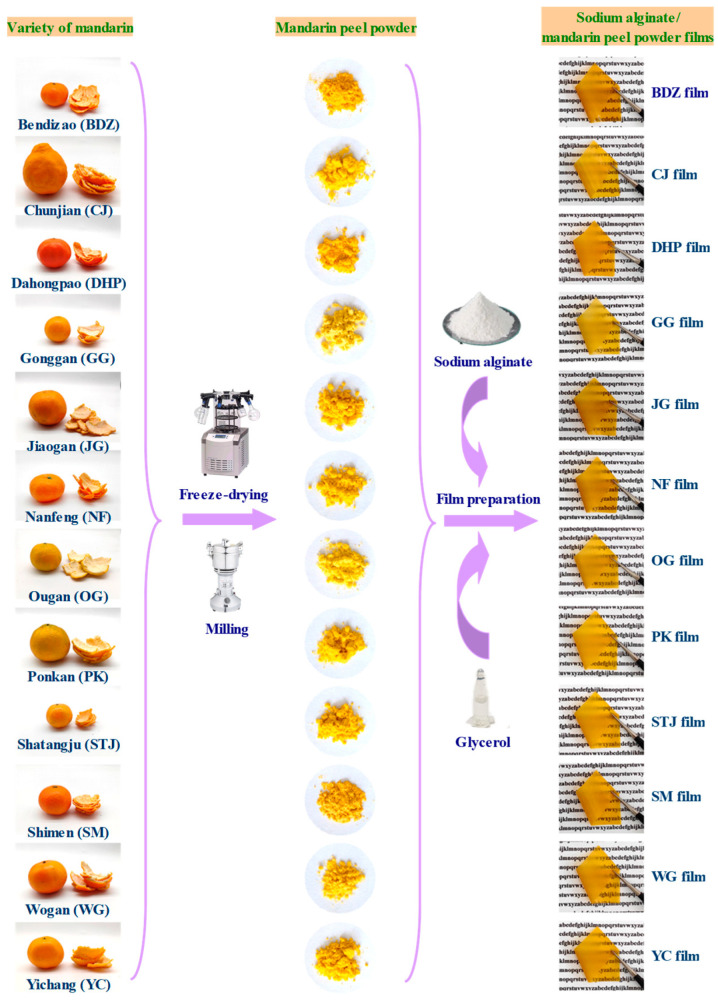
Flow chart for the production of active food packaging films based on sodium alginate and twelve varieties of mandarin peel powder.

Fresh mandarin fruits were washed and peeled. Each variety of mandarin peel was individually collected, freeze-dried, and milled into 100-mesh powder (Figure 1). The proximate composition of the mandarin peel powders, including ash, pectin, protein, lipid, crude fiber, and total polyphenol contents, were analyzed according to Yun et al. [23].

### 2.3. Preparation of Active Packaging Films

Firstly, mandarin peel powder suspension was prepared by homogenizing 4.6 g peel powder in 170 mL water at 6000 rpm for 2 min. Then, sodium alginate (1.38 g) and glycerol (1.38 g) were thoroughly mixed with the mandarin peel powder suspension at 20 °C for 1 h. The whole mixture was poured into a square polymethyl methacrylate plate with a boundary length of 25 cm and then dried at 35 °C for 36 h [23]. Based on the variety of mandarin, the obtained films were named BZD, CJ, DHP, GG, JG, NF, OG, PK, STJ, SM, WG, and YC (Figure 1).

### 2.4. Structural Characterization of Films

#### 2.4.1. Scanning Electron Microscopy (SEM)

Cross-sectional images of the gold-sputtered film specimens were taken using GeminiSEM 300 scanning electron microscopy (Carl Zeiss Cop., Oberkochen, Germany) at 5 kV.

#### 2.4.2. Infrared Spectroscopy

The film specimens were scanned between 4000 and 400 cm^−1^ under a Varian 670 infrared spectrometer (Varian Corp., Palo Alto, CA, USA) at 4 cm^−1^ resolution.

#### 2.4.3. X-ray Diffraction (XRD)

The film specimens were subjected to XRD analysis under a D8-Advance diffractometer (Bruker-AXS GmbH, Karlsruhe, Germany) ranging between 5° and 75°. The crystalline degree of the film specimen was determined using Jade 6.0 software (Material Date Inc., Livermore, CA, USA).

### 2.5. Determination of the Physical and Functional Properties of Films

#### 2.5.1. Optical Properties

The optical properties of the film specimens, including color and light transmittance, were determined using the method by Huang et al. [24]. First, an SC-80C colorimeter (Kangguang Corp., Beijing, China) was applied to measure the L*, a*, b*, and Δ*E* of each film specimen, where L*, a*, b*, and Δ*E* individually represented lightness, redness, yellowness, and total color difference, respectively. Afterwards, a Lambda 35 UV-vis spectrophotometer (PerkinElmer Corp., Waltham, MA, USA) was employed to test the light transmittance of film specimens, ranging between 200 and 800 nm.

#### 2.5.2. Thickness

A micrometer (EVERTE, Bonthe Corp., Shangqiu, China) was employed to measure the thickness of film specimens.

#### 2.5.3. Water Blocking Ability

The water blocking ability of film specimens was evaluated in terms of moisture content (MC), water contact angle (WCA), and water vapor permeability (WVP), according to Yun et al. [23]. Briefly, MC was calculated based on the gravimetric change of film specimens after thoroughly drying at 105 °C. WCA was determined by recording the image of a water drop (2 μL) on the film surface and calculating the contact angle between the water drop and the film with a GP-50 analyzer (Gaopin Corp., Suzhou, China). WVP was tested by placing a film specimen-mounted tube (50 mL) in an airtight container at 20 °C with 100% relative humidity. The tube was filled with 16 g dried silica gel and the gravimetric change of the tube over 4 days was used to calculate WVP. The WVP of the film was calculated as follows:

(1) $$\mathrm{WVP}=\frac{W\times x}{t\times A\times \Delta P}$$

where *W* was the increased weight of the tube (g), *x* was the film thickness (m), *t* was the time (s) for the weight gain of the tube, *A* was the permeable area (m^2^) of water vapor, and Δ*P* was the saturated vapor pressure at 20 °C.

#### 2.5.4. Oxygen Permeability (OP)

The OP of the film specimens was tested using a Basic 201 gas permeability analyzer (Labthink Corp., Jinan, China) at 50% relative humidity and 20 °C [25]. The OP of each film was calculated as follows:

(2) $$\mathrm{OP}=\frac{OTR\times x}{\Delta P}$$

where *OTR* was the oxygen transmission rate (m s^−1^), *x* was the film thickness (m), and Δ*P* was the pressure difference between the two compartments of the gas permeability analyzer (Pa).

#### 2.5.5. Mechanical Properties

The mechanical properties of each film specimen were tested on a universal tester (STX200, Yishite Corp., Xiamen, China) with a stretching speed of 6 cm/min. Tensile strength (TS) and elongation at break (EB) were recorded when the film fractured [26].

#### 2.5.6. Thermogravimetric Analysis (TGA)

TGA was carried out using an HTG-1 tester (Henven Corp., Beijing, China) by heating each film specimen (about 2 mg) from 50 to 750 °C (10 °C min^−1^) under nitrogen flow (20 mL min^−1^) and recording the thermogravimetry (TG) curve. The derivative thermogravimetry (DTG) curve was obtained via the first-order derivative of TG curve.

#### 2.5.7. Antioxidant-Releasing Ability

To test antioxidant-releasing ability, a rectangular film specimen (1 cm × 4 cm) was soaked in 5 mL of 95% ethanol with shaking at 120 rpm for 1 h. The total phenol content (TPC) and the released antioxidant activity in the solvent were determined by reacting with Folin-Ciocalteau and DPPH reagents, respectively, according to Yun et al. [23]. The TPC released from the film was determined by reacting 1 mL of film specimen solution with 1 mL of Folin-Ciocalteau reagent. The released antioxidant activity was measured by reacting 1 mL supernatant with 3 mL DPPH methanol solution in the dark for 1 h. Results were recorded as milligram gallic acid equivalent (GAE) per g film for the TPC test, and μmol Trolox equivalent (TE) per g film for the DPPH radical scavenging test.

#### 2.5.8. Antimicrobial-Releasing Ability

To test antimicrobial-releasing ability, a rectangular film specimen (1 cm × 4 cm) was soaked in a bacterial suspension of *Staphylococcus aureus* ATCC 6538 and *Escherichia coli* ATCC 43895 (10^6^ CFU mL^−1^), followed by incubation at 37 °C for one day. The obtained bacterial culture (100 μL) was gradient-diluted and incubated on a nutrient broth agar plate at 37 °C for another day. The colonies formed on the plate were counted. Control experiments were conducted following the same procedure, excepting the addition of the film specimen to bacterial suspension [27].

#### 2.5.9. Biodegradability

To test biodegradability, square film specimens (2.5 cm × 2.5 cm) were placed on aluminum mesh to allow microbial action and moisture transfer and buried in 7 cm of moist soil at 20 °C for 25 days. The soil was sprayed with distilled water once a day to maintain soil moisture. The morphological change and weight loss of each film specimen were recorded at an interval of 5 days, according to Ahmad et al. [16].

### 2.6. Corn Oil Packaging Test of Films

The anti-permeability of each film specimen was evaluated by covering film specimens (4.5 cm × 4.5 cm) over the open mouth of a test tube [17]. The test tube, containing 10 mL corn oil, was inverted upon filter paper. If oil permeated across the film, oil could be observed on the filter paper. The anti-permeability test was conducted at 25 °C for 15 days. Thereafter, the corn oil packaging test was conducted by storing 5 mL oil in a heat-sealed film sachet (7 cm × 7 cm) at 50 °C for 10 days [28]. The oxidation degree of the oil was evaluated by measuring the peroxide value (PV) and thiobarbituric acid reactive substance (TBARS) at an interval of 2 days [29].

### 2.7. Statistical Analysis

Data were analyzed by SPSS 20.0 software (SPSS Inc., Chicago, IL, USA) using one-way analysis of variance followed by Duncan test (*p* < 0.05). The correlations between the component content of the mandarin peel powders and the structures and properties of the films were determined by Pearson analysis.

## 3. Results and Discussion

### 3.1. Proximate Composition of Mandarin Peel Powder

As shown in Figure 1, the twelve varieties of fresh mandarin fruits had different sizes and colors. Peels were removed from mandarin fruits, freeze-dried, and milled into powder. Table 1 summarizes the proximate composition for twelve varieties of mandarin peel powder. The mandarin peel powders contained pectin (12.27–18.43%), lipid (7.73–13.58%), protein (4.17–7.90%), crude fiber (3.32–5.00%), ash (2.81–4.00%), and total phenol (4.97–6.70 mg GAE g^−1^). The component contents of the mandarin peel powders were comparable to several previous studies [23,30,31]. Results indicated the proximate composition of the mandarin peel powders was influenced by mandarin variety, with CJ and NF having the highest pectin content, CJ having the highest protein content, NF having the highest lipid content, SM having the highest crude fiber content, and CJ having the highest TPC. Some previous studies have reported that different varieties of citrus fruit exhibit varying compositions of pectin, crude fiber, lipid, and polyphenols in the respective fruit peel powder [32,33,34,35,36]. Liu et al. [35] documented that pectin isolated from different varieties of citrus fruit peel powder had different monosaccharide compositions and molecular weights. Wang et al. [36] found that the insoluble crude fiber content (e.g., cellulose, hemicellulose, and lignin) of citrus fruit peel powders was affected by the variety of citrus fruit. Hosni et al. [34] demonstrated that limonene was the key element in the essential oils of different citrus fruit peels. Anticona et al. [32] suggested that hesperidin and narirutin were the most abundant polyphenols in different varieties of mandarin peel powder. In this study, different varieties of mandarin peel powder were further used to prepare active food packaging films (Figure 1).

### 3.2. Structural Characteristics of Films

**Figure 2:**
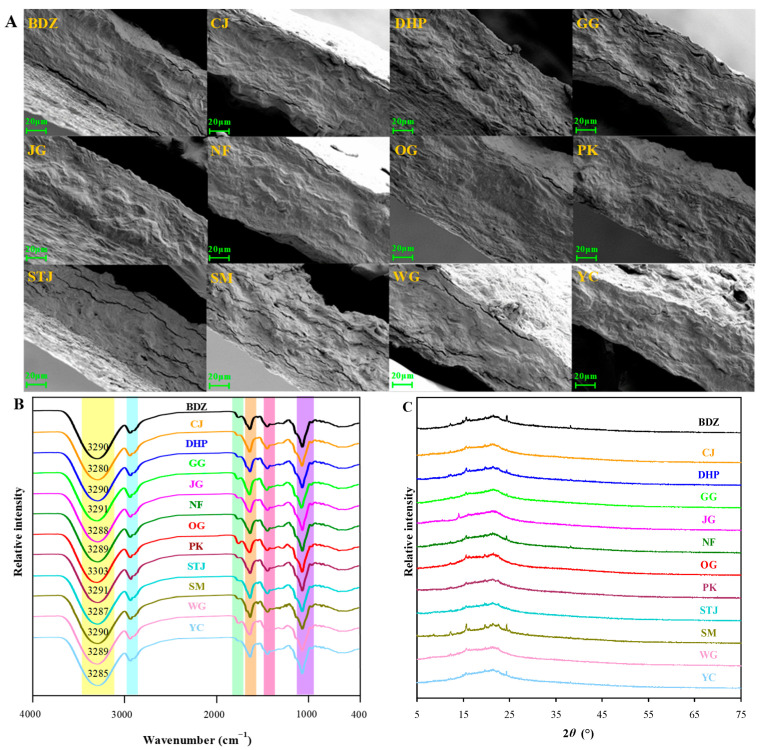
Cross-sectional morphologies (**A**), infrared spectra (**B**), and XRD pattern (**C**) of sodium alginate/mandarin peel powder films.

Figure 2 shows the structural characteristics of sodium alginate/mandarin peel powder films, which were characterized by SEM, infrared spectrometry, and use of an X-ray diffractometer. The prepared films showed different cross-sectional morphologies (Figure 2A). Amongst the twelve films, the CJ, JG, NF, OG, PK, and YC films showed compact cross-sections without obvious inner cracks. In contrast, the BDZ and DHP films had some cracks inside. The situation was much worse for the GG, STJ, SM, and WG films. The morphological differences in the films could be explained by the compositional content of the different mandarin peel powders (Table 1). The CJ, JG, NF, OG, PK, and YC peel powders had higher contents of pectin, protein, and lipid, which may have interacted with each other to form the tightly packed films [37]. However, BDZ, DHP, GG, STJ, SM, and WG peel powder had higher contents of insoluble crude fiber (4.08–5.00%). The insoluble crude fiber had low compatibility with other film components, thereby disrupting the integrity of the films. The cracks in the BDZ, DHP, GG, STJ, SM, and WG films were adverse to the barrier and mechanical properties of these films. Recently, Yun et al. [23] also found that citrus fruit peel powders with higher contents of crude fiber were disadvantageous in the production of dense packaging films.

Although different varieties of mandarin peel powder had different component contents, the sodium alginate/mandarin peel powder films showed similar infrared spectra (Figure 2B). A broad peak (O–H/N–H stretching) around 3300 cm^−1^ was caused by hydroxyl groups in all film components, as well as amino groups in protein [17,38]. Notably, different films showed some differences in the position of this infrared peak, varying from 3280 to 3303 cm^−1^. This was because the different varieties of mandarin peel powder had different component contents (Table 1), which could have influenced the hydrogen bonds of film components [23]. The films displayed double peaks (C–H stretching) around 2925 cm^−1^, attributed to methylene-containing components (e.g., pectin, protein, crude fiber, sodium alginate, and glycerol) [18,39]. The films also showed characteristic ester carbonyls around 1735 cm^−1^, which are present in pectin, lipids, and sodium alginate [19]. The peak around 1600 cm^−1^ (C=C stretching) corresponded to polyphenols [40]. Other peaks around 1410 and 1025 cm^−1^ corresponded to CH–CH_2_ bending and C–O–C stretching of film components, respectively, which are frequently observed in fruit peel-based packaging films [38,41]. The above results suggest that the the infrared spectra of the films were only minorly influenced by the variety of mandarin peel powder.

**Figure 3:**
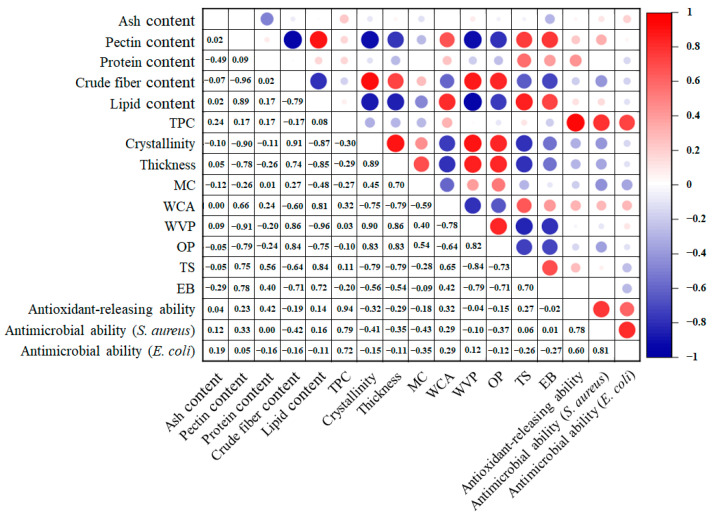
Correlation analysis between the component content of mandarin peel powder and the structural, physical, and functional properties of sodium alginate/mandarin peel powder films. Circles of different sizes indicate the magnitude of the correlation.

Figure 2C shows the XRD patterns of the sodium alginate/mandarin peel powder films. Several crystalline peaks were observed in these films, ranging from 9° to 40°. The sharp crystalline peaks were attributed to the crude fiber (e.g., cellulose, hemicellulose, and lignin) in mandarin peel powder, which have also been observed in other biopolymer/fruit peel powder packaging films, such as starch/pomegranate peel powder film and chitosan/pomegranate peel powder film [42,43]. The results revealed that the sodium alginate/mandarin peel powder films were semi-crystalline. The crystalline degree of the films varied from 5.49% to 13.91%, indicating that the variety of mandarin peel powder used had a major impact on the crystallinity of the films. Correlation analysis was then performed between the component content of the mandarin peel powders and the crystalline degree of the films. As shown in Figure 3, the crude fiber content of mandarin peel powder had a highly positive correlation with the crystalline degree of the films (*R*^2^ = 0.91), verifying the major contribution of crude fiber to the semi-crystalline status of the films. Conversely, the pectin and lipid contents of mandarin peel powder showed highly negative correlations with the crystalline degree of the films (*R*^2^ = −0.90 for pectin content–crystalline degree correlation, and *R*^2^ = −0.87 for lipid content–crystalline degree correlation). This was probably because pectin and lipid were the two main components in mandarin peel powders with amorphous natures. Pectin and lipid could interact with crude fiber through hydrogen bonds, resulting in a decreased crystalline degree of the films. Some researchers have also found that the interactions between pectin and crude fiber decreased the degree of crystallinity of the films [44].

### 3.3. Optical Properties of Films

All of the sodium alginate/mandarin peel powder films were yellow (Figure 1), similar to the color of raw mandarin peel powder. This was because mandarin peel powder was the main component of the films, and the other film components (e.g., sodium alginate and glycerol) were almost colorless. The yellow color of the films was primarily caused by natural pigments in mandarin peel powder, especially carotenoids and polyphenols [45]. Table 2 shows that all the films had very high b* values, varying from 64.99 to 78.35. This further demonstrates the yellowness of the films. On one hand, the color of the films was related to the carotenoid and polyphenol contents of mandarin peel powder, as intuitively reflected by the color of mandarin peel powder (Figure 1). On the other hand, the color of the films was influenced by the ratio of the outer yellow flavedo layer and the inner white albedo layer of mandarin peels [46]. Since GG, OG, and WG peels had relatively thicker white albedo layers (Figure 1), the produced GG, OG, and WG films exhibited relatively lower b* values than other films. 

**Figure 4:**
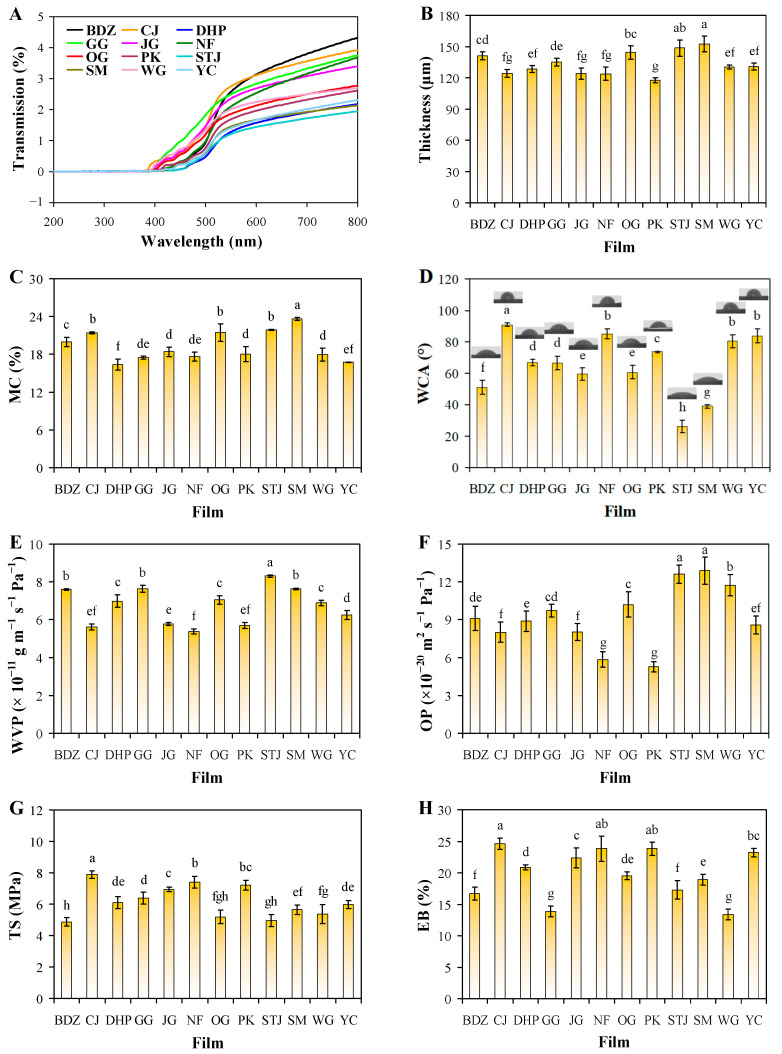
Light transmittance (**A**), thickness (**B**), MC (**C**), WCA (**D**), WVP (**E**), OP (**F**), TS (**G**), and EB (**H**) of sodium alginate/mandarin peel powder films. Data are given as mean ± SD (*n* = 10 for thickness, *n* = 3 for MC, WVP and OP, *n* = 6 for WCA, TS and EB). The inserted pictures in (**D**) show the WCA of different films. Different lower case letters indicate significant difference (*p* < 0.05). MC: moisture content; WCA: water contact angle; WVP: water vapor permeability; OP: oxygen permeability; TS: tensile strength; EB: elongation at break.

Figure 1 shows that all the films were transparent. Despite this, the light transmittance of the films was smaller than 5% at visible light range and almost zero at UV light range, revealing that the films had strong UV-vis light blocking performance (Figure 4A). This was because the films contained crude fiber that could block light transmittance [20]. Meanwhile, many film components including pectin, protein, lipid, lignin, sodium alginate and polyphenols contained unsaturated bonds that could absorb UV light [23]. By contrast, sodium alginate/mandarin peel powder films showed lower light transmittance than previously prepared pomelo peel flour films [17], chitosan/polyvinyl alcohol/orange peel composite films [20], and different citrus fruit peel powder-based films [23].

### 3.4. Thickness and Water Blocking Ability of Films

As the different varieties of mandarin peel powder had different component contents, the produced sodium alginate/mandarin peel powder films showed thickness values ranging from 117.73 to 152.45 μm (Figure 4B). Notably, the thickness of the films displayed a positive correlation with the crude fiber content in the mandarin peel powder, but negative correlations with the pectin and lipid contents in mandarin peel powder (Figure 3). This result agreed with SEM observations (Figure 2A), showing that the films with high crude fiber contents had loosely packed structures, whereas the films with high pectin and lipid contents had tightly packed structures. Amongst the films, PK film exhibited the lowest thickness, as the PK peel powder had high pectin and lipid contents, but the lowest crude fiber content. On the contrary, the SM film displayed the greatest thickness, as the SM peel powder had the highest crude fiber content, but the lowest pectin and lipid contents. The sodium alginate/mandarin peel powder films showed similar thickness compared to previously prepared chitosan/polyvinyl alcohol/orange peel composite films with 120–138 μm of thickness [20], had lower thickness than mosambi peel/sago powder films with 0.34–0.70 mm of thickness [16], but had higher thickness than pomelo peel flour films with 80.6–101.0 μm of thickness [17], and pomelo peel flour films with 60.4 μm of thickness [18].

Sodium alginate/mandarin peel powder films showed MC from 16.39% to 23.62% (Figure 4C). It is worth noting the MC of the films was positively correlated with their thicknesses (Figure 3). This further revealed that the moisture in the films greatly contributed to thickness enhancement. However, the MC of the films did not show high correlations with the component content in mandarin peel powder. The SM film presented the highest MC because the films had several inner cracks (Figure 2A) that could retain moisture. In another study, Meydanju et al. [39] also found that the MC of lemon peel powder-based films was related with the compactness of the films. The MC of the films decreased after the voids in the films were filled with metal nanoparticles. The sodium alginate/mandarin peel powder films had similar MC to previously prepared pomelo peel flour films with 15.14–19.72% MC [17], but had higher MC than mosambi peel/sago powder films with 10.00–14.67% MC [16], pomelo peel flour film with 14.84% MC [18], and gelatin/orange peel powder films with 11.30–12.67% MC [21].

Sodium alginate/mandarin peel powder films had WCA ranging from 26.03° to 90.75° (Figure 4D). This indicates that the variety of mandarin peel powder used had a major impact on the surface wettability of the films. Figure 3 shows that the WCA of the films had a positive correlation with the lipid content in the mandarin peel powder (*R*^2^ = 0.81), demonstrating that lipid content was beneficial in increasing the hydrophobicity of the films. This is due to the fact that lipids are hydrophobic substances that can act as a good water barrier [47]. Notably, the STJ and SM films showed relatively lower WCA than other films due to the fact that these two films had not only lower lipid contents, but also higher crude fiber contents (Table 1). Crude fiber could disrupt the compactness of the films and produced loosely packed structures (Figure 2A), which increased the wettability of the films. In this respect, the WCA of the films had a negative correlation with the crude fiber content in mandarin peels (*R*^2^ = −0.60), as displayed in Figure 3. The sodium alginate/mandarin peel powder films showed similar WCA to previously prepared poly(lactic acid)/orange peel powder films with 70.12–88.18° WCA [22], had overwhelmingly higher WCA than pomelo peel flour films with 32.37–42.03° WCA [17], but had lower WCA than chitosan/polyvinyl alcohol/orange peel composite films with 78.38–104.7° WCA [20].

WVP is often used to evaluate the moisture blocking ability of packaging films. Sodium alginate/mandarin peel powder films had WVP between 5.38 × 10^−11^ and 8.31 × 10^−11^ g m^−1^ s^−1^ Pa^−1^ (Figure 4E). Figure 3 shows that the WVP of the films was negatively correlated with their WCA (*R*^2^ = −0.78), demonstrating that the films with high surface wettability also had low moisture blocking ability. Notably, the WVP of the films was positively correlated with the crude fiber content in the mandarin peel powder (*R*^2^ = 0.86), with the STG film having the highest WVP. This is because crude fiber creates cracks within the films, which helps moisture to quickly pass through the films. On the contrary, the WVP of the films was negatively correlated with the contents of pectin and lipid in mandarin peel powder, because pectin and lipids are beneficial in producing films with dense structures (Figure 2A). At the same time, the hydrophobic character of lipids could also contribute to the moisture blocking ability of the films [47]. Many research groups have also demonstrated that the compactness of citrus fruit peel powder-based films has a large impact on their WVP [21,23,39]. The sodium alginate/mandarin peel powder films had similar WVP to previously prepared lemon waste powder film with 6.83 × 10^−11^ g m^−1^ s^−1^ Pa^−1^ WVP [19], but had lower WVP than pomelo peel flour films with 20.8 × 10^−11^–25.8 × 10^−11^ g m^−1^ s^−1^ Pa^−1^ WVP [17] and pomelo peel flour film with 23.3 × 10^−11^ g m^−1^ s^−1^ Pa^−1^ WVP [18]. 

### 3.5. Oxygen Blocking Ability of Films

The sodium alginate/mandarin peel powder films had OP from 5.26 to 12.91 × 10^−20^ m^2^ s^−1^ Pa^−1^, reflecting the oxygen blocking ability of the films (Figure 4F). The OP of the films showed a positive correlation with their WVP (Figure 3). Moreover, the OP of the films was negatively correlated with the contents of pectin and lipid in mandarin peel powder but positively correlated with the crude fiber content in mandarin peel powder. The above results suggest that the moisture and oxygen blocking abilities of the films had similar trends, and both of them were associated with the inner structures of the films. Amongst the films, the NF and PK films had the highest oxygen blocking ability, as the NF and PK peels had high pectin and lipid contents but the lowest crude fiber contents, presenting dense structures (Figure 2A). On the contrary, the SM film exhibited the lowest oxygen blocking ability because the SM peel had the lowest pectin and lipid contents but the highest crude fiber content, displaying a cracked and loosely packed structure. Terzioğlu et al. [20] and Yun et al. [23] also reported that citrus fruit peel powder-based films with tightly packed structures had good oxygen blocking ability. The sodium alginate/mandarin peel powder films showed similar OP as previously prepared different citrus fruits peel powder based films with 4.11–7.88 × 10^−20^ m^2^ s^−1^ Pa^−1^ OP [23], but had lower OP than chitosan/polyvinyl alcohol/orange peel composite films with 34.52–37.01 × 10^−20^ m^2^ s^−1^ Pa^−1^ OP [20].

### 3.6. Mechanical Properties of Films

Figure 4G,H show that the TS and EB of the sodium alginate/mandarin peel powder films were 4.87–7.90 MPa and 13.37–24.62%, respectively. Figure 3 reveals that the TS and EB of the films had similar trends, presenting a positive correlation between them (*R*^2^ = 0.70). The TS and EB of the films had positive correlations with the contents of pectin and lipid in the mandarin peel powders, but negative correlations with the crude fiber content in the mandarin peel powders. The mechanical properties of the films were consistent with their WVP (Figure 4E) and OP (Figure 4F), which were all related to the inner structures of the films (Figure 2A). The CJ, NF, and PK films, with higher pectin and lipid contents, had dense structures that could withstand mechanical force, resulting in stronger mechanical properties. However, the BDZ, GG, STG, SM, and WG films, with higher crude fiber contents, had cracked structures and presented weaker mechanical properties. Similarly, Yun et al. [23] found that the mechanical properties of citrus peel powder-based films was negatively correlated with the WVP and OP of the films. By contrast, the sodium alginate/mandarin peel powder films showed similar TS as compared to previously prepared lemon waste powder film with 7.64 MPa TS [19], but had lower TS than pomelo peel flour films with 13.75–23.55 MPa TS [17], pomelo peel flour film with 17.52 MPa TS [18], chitosan/polyvinyl alcohol/orange peel composite films with 17.77–21.29 MPa TS [20], and gelatin/orange peel powder films with 23.21–27.22 MPa TS [21]. The sodium alginate/mandarin peel powder films showed similar EB as previously prepared pomelo peel flour film with 19.46% EB [18] and lemon waste powder film with 24.24% EB [19], slightly higher EB than pomelo peel flour films with 9.31–17.68% EB [17], and lower EB than chitosan/polyvinyl alcohol/orange peel composite films with 234.67–257.52% EB [20] and gelatin/orange peel powder films with 42.25–61.69% EB [21].

### 3.7. Thermal Properties of Films

TG and DTG curves for the sodium alginate/mandarin peel powder films showed that the films had four decomposition processes (Figure S1). As summarized in Table S1, the first process, with weight loss between 5.19% and 15.57%, was caused by water vaporization. The second process, with weight loss between 36.31% and 46.09%, was the main decomposition stage, which was attributed to the pyrolysis of glycerol, sodium alginate, pectin, protein, cellulose, and hemicellulose [23,48]. During the second process, the maximum decomposition rate of the films appeared at 183–203 °C. The third process, with weight loss between 9.49% and 16.45%, was mainly due to the pyrolysis of cellulose [48]. The fourth process was associated with the decomposition of lignin and carbonaceous residues [48]. When the temperature reached 750 °C, the solid residues of the PK and NF films were still higher than 15%. Based on the TG curves, it could be concluded that the PK, NF, JG, and CJ films were more stable than other films. This was consistent with the results regarding WVP (Figure 4E), OP (Figure 4F), and TS (Figure 4G), which were all attributed to the inner structures of these films. The four abovementioned films had dense structures and could withstand thermal treatment.

### 3.8. Antioxidant-Releasing Ability of Films

**Figure 5:**
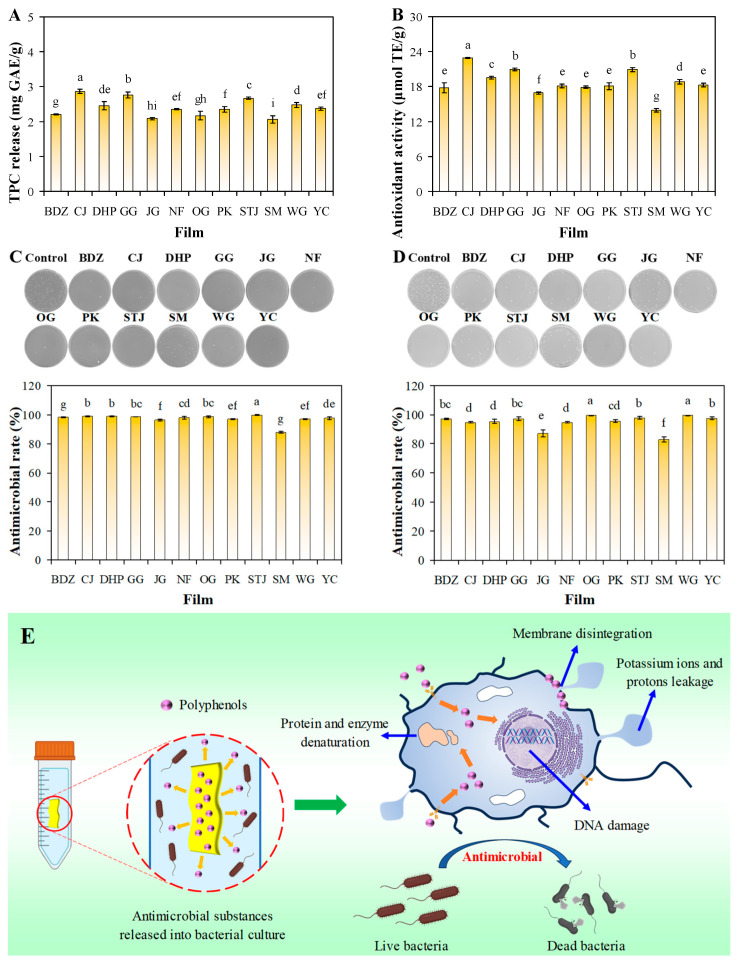
TPC (**A**), DPPH radical scavenging activity (**B**), antimicrobial-releasing ability against *S. aureus* (**C**) and *E. coli* (**D**), and antimicrobial mechanisms (**E**) of sodium alginate/mandarin peel powder films. Data are given as mean ± SD (*n* = 3). Different lower case letters indicate significant difference (*p* < 0.05).

Antioxidant-releasing ability is an important functional property for active packaging films as it aids in the retardation of food oxidation. The antioxidant-releasing ability of the sodium alginate/mandarin peel powder films was tested in 95% ethanol, simulating the antioxidant behavior of the films in fatty food [49]. Figure 5A,B show the TPC and DPPH radical scavenging activity of the films, respectively. After the films were shaken in 95% ethanol for 1 h, the films released 2.06–2.86 mg GAE g^−1^ total phenol. Meanwhile, the DPPH radical scavenging activity of the released polyphenols reached 13.94–22.91 μmol TE g^−1^. Figure 3 shows that the antioxidant-releasing abilities of the films were highly positive correlated with the TPC in mandarin peels (*R*^2^ = 0.94), revealing that the polyphenols in mandarin peel powder were responsible for the antioxidant behavior of the films. Amongst the films, the CJ film showed the highest antioxidant-releasing ability, which was 1.64 times that of the SM film, which had the lowest antioxidant-releasing ability. Other researchers have also recently reported that citrus fruit peel powder-based films have good antioxidant-releasing ability [20,21,23]. Except for TPC in mandarin peel powder, the other components in mandarin peels had little impact on the antioxidant-releasing ability of the films. This indicates that the antioxidant-releasing ability of the films was not affected by their inner structures. In other words, all the films effectively released polyphenols into the simulated fatty food.

### 3.9. Antimicrobial-Releasing Ability of Films

The antimicrobial-releasing ability of the sodium alginate/mandarin peel powder films was tested in a liquid culture medium containing *S. aureus* and *E. coli*. The film-treated bacterial suspension was cultured in a solid culture medium, and the formed colonies were counted and used to calculated the antimicrobial rate of the films. As shown in Figure 5C,D, more than two hundred colonies formed on the control plates without film treatment. However, the growth of *S. aureus* and *E. coli* was significantly inhibited by the films, with most plates presenting less than twenty colonies. The antimicrobial rate of the films ranged from 83.02% to 99.77%, indicating that the sodium alginate/mandarin peel powder films had strong antimicrobial-releasing ability. Other researchers have also demonstrated that mandarin peel powder-based films could inhibit the growth of *S. aureus* and *E. coli* [23,50]. Notably, the antimicrobial rate of the films against *S. aureus* and *E. coli* showed positive correlations with the TPC in mandarin peels (Figure 3), revealing that polyphenols played a main role in inhibiting microbial growth. Similar to the antioxidant-releasing ability of the films, the antimicrobial-releasing ability of the films was not affected by their inner structures. Based on the literature [51,52,53,54], the antimicrobial mechanisms of the films are illustrated in Figure 5E. The antimicrobial agents (i.e., polyphenols) were released from the films and could interfere with or inhibit microbial growth and proliferation in different ways, such as increasing the permeability or disintegration of microbial membranes, inducing the leakage of potassium ions and protons from microbial cells, denaturing protein and enzyme activity, and damaging DNA molecules.

### 3.10. Biodegradability of Films

**Figure 6:**
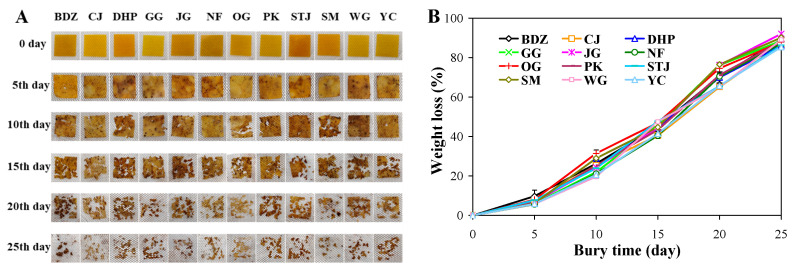
Morphological change (**A**) and weight loss (**B**) of sodium alginate/mandarin peel powder films buried in moist soil at 20 °C for 25 days. Data are given as mean ± SD (*n* = 3).

Citrus fruit peel powder-based packaging films, due to their biodegradable nature, have been considered as alternatives to synthetic plastics [15]. Although many studies have focused on citrus fruit peel powder-based films, the biodegradability of the films has seldom been evaluated [22]. In this study, sodium alginate/mandarin peel powder films were buried in moist soil, and the morphological changes in, and weight loss of, the films were monitored. As displayed in Figure 6, all the films were gradually degraded into debris. The biodegradable nature of the films was reflected by observed weight loss, which progressively increased over the course of 25 days. At the end of 25th day, the weight loss of these films reached 85.39–92.01%, which was higher than the reported poly(lactic acid) films containing orange peel powder [22]. This suggests that all the films had good biodegradability. As reported, the biodegradation of bio-based packaging films normally comprises three main stages: (1) the colonization of soil microorganisms on the film surface, (2) the depolymerization of film components under the action of microbial enzymes, and (3) the utilization of film-degraded products as microbial nutrients, with the release of water and CO_2_ [55]. The findings of this study revealed that the biodegradability of the sodium alginate/mandarin peel powder films was not particularly affected by mandarin variety. The biodegradable nature of the films makes it possible to utilize mandarin peels in a renewable and sustainable way.

### 3.11. Application of Films

**Figure 7:**
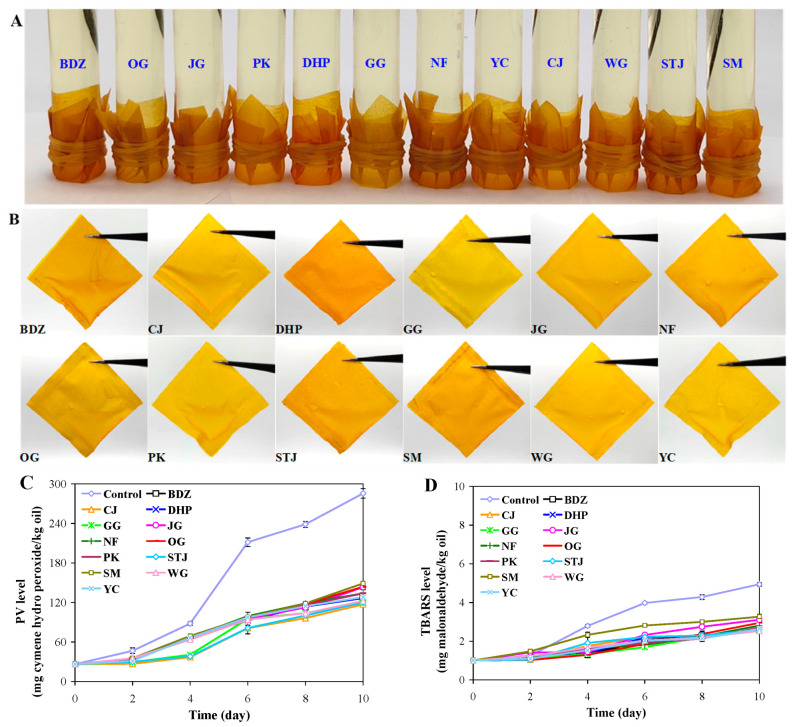

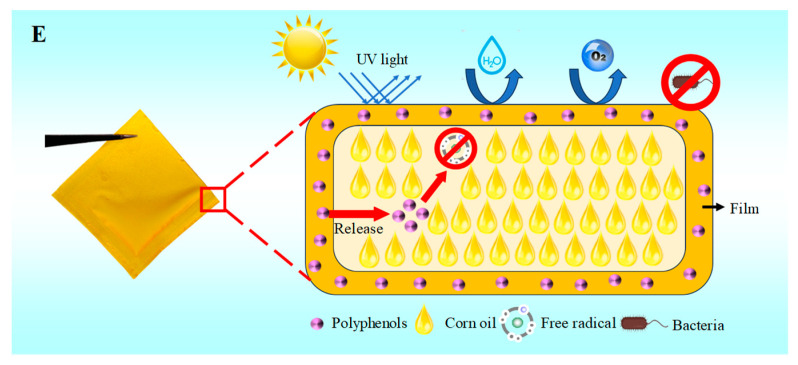
Anti-permeability test of sodium alginate/mandarin peel powder films against corn oil (**A**), film sachets for packaging corn oil (**B**), PV (**C**) and TBARS (**D**) levels of corn oil in film sachets at 50 °C for 10 days, and the protective mechanisms of film sachets on corn oil (**E**). Data are given as mean ± SD (*n* = 3).

Edible oil is an indispensable nutritional resource in the human diet. However, several environmental factors including heat, light, oxygen, and moisture can trigger the oxidation of oil during storage [56]. In recent years, researchers have preferred to use natural antioxidants to elevate the oxidative stability of oil, because they are much safer than synthetic antioxidants [57]. Considering that the sodium alginate/mandarin peel powder films had good antioxidant-releasing ability in simulated fatty food (Figure 5A,B), they were used to package corn oil. Beforehand, an anti-permeability test was performed to verify whether oil could leak from the films (Figure 7A). After being tested for 15 days, all of the films showed good anti-permeability against corn oil. Thus, the films were further processed into small sachets for corn oil packaging (Figure 7B). The oxidation degree of oil was evaluated by PV and TBARS values, individually corresponding to the primary and secondary oxidative products of oil. As shown in Figure 7C, the PV level of the control oil rapidly increased at day 4–10. By contrast, the TBARS level of control oil slowly increased over the course of 10 days. This was because heat, light, oxygen, and moisture had bigger impacts on the initial stage of oil oxidation [58]. As compared with the control oil, the oil packaged in film sachets showed lower PV and TBARS levels. At the end of 10 days, the PV and TBARS levels of sachet-packaged oil were reduced by 47.83–58.98% and 33.81–48.79%, respectively, in comparison with those of the control oil. Results revealed that the sodium alginate/mandarin peel powder films effectively delayed oil oxidation. Notably, these films showed some differences in delaying oil oxidation, because the films had different physical and functional properties. The protective mechanisms of the films on corn oil were proposed in Figure 7E. As demonstrated above, the sodium alginate/mandarin peel powder films had good barrier abilities against light, moisture, and oxygen (Figure 4), which are key factors triggering oil oxidation. At the same time, polyphenols were slowly released from the sodium alginate/mandarin peel powder films, which could have played a role in quenching free radicals involved in oil oxidation (Figure 5). In addition, polyphenols in the films acted as an antimicrobial barrier against external microorganisms, which could further improve the safety of oil.

## 4. Conclusions

This study compared the structures and functional properties of active food packaging films based on sodium alginate and twelve varieties of mandarin peel powder. All the films showed superior light barrier ability, antimicrobial-releasing ability, and biodegradability. The CJ, NF, and PK films showed better moisture/oxygen barrier abilities and mechanical properties. The CJ, GG, STJ, and WG films exhibited better antioxidant-releasing ability and oil oxidation inhibitory ability. Considering all of the properties of the films, the CJ film had the optimal performance. Moreover, this study analyzed the correlations between the component content of mandarin peel powder and the structures and properties of the films. The structures and physical properties of the films were positively correlated with the pectin and lipid contents in mandarin peel powder, and were negatively correlated with the crude fiber content in mandarin peel powder. The antioxidant-releasing and antimicrobial-releasing abilities of the films were positively correlated with the TPC in the mandarin peel powders. In the future, further studies are needed to improve the mechanical properties of the films for practical applications. Additionally, factors affecting consumer acceptance of the films (e.g., their color and aroma) should be assessed before the films are released to market.

## Figures and Tables

**Table 1:** Proximate composition for twelve varieties of mandarin peel powder.

Variety	Ash (%)	Pectin (%)	Protein (%)	Crude Fiber (%)	Lipid (%)	TPC (mg GAE/g)
BDZ	4.00 ± 0.16 ^b^	14.24 ± 0.57 ^de^	4.44 ± 0.50 ^fg^	4.08 ± 0.79 ^cd^	8.72 ± 0.11 ^fg^	6.08 ± 0.16 ^bc^
CJ	3.05 ± 0.31 ^e^	18.13 ± 0.79 ^a^	7.90 ± 0.13 ^a^	3.52 ± 0.23 ^e^	12.82 ± 0.74 ^b^	6.70 ± 0.14 ^a^
DHP	2.82 ± 0.16 ^e^	14.75 ± 0.57 ^cd^	6.75 ± 0.50 ^b^	4.43 ± 0.28 ^bc^	10.43 ± 0.40 ^e^	6.10 ± 0.02 ^bc^
GG	3.87 ± 0.25 ^bc^	12.37 ± 0.64 ^f^	7.01 ± 0.63 ^b^	4.72 ± 0.22 ^ab^	9.26 ± 0.08 ^f^	6.44 ± 0.24 ^ab^
JG	2.98 ± 0.11 ^e^	16.77 ± 0.71 ^b^	5.33 ± 0.25 ^de^	3.64 ± 0.17 ^de^	12.51 ± 0.29 ^b^	5.58 ± 0.08 ^d^
NF	4.74 ± 1.48 ^a^	18.43 ± 0.50 ^a^	4.79 ± 0.63 ^ef^	3.32 ± 0.28 ^e^	13.58 ± 0.39 ^a^	5.95 ± 0.01 ^cd^
OG	2.81 ± 0.26 ^e^	14.85 ± 0.36 ^cd^	5.41 ± 0.37 ^d^	4.00 ± 0.34 ^cd^	9.16 ± 0.16 ^f^	5.70 ± 0.39 ^cd^
PK	3.22 ± 0.11 ^de^	16.81 ± 0.64 ^b^	6.84 ± 0.63 ^b^	3.52 ± 0.34 ^e^	11.17 ± 0.11 ^d^	5.96 ± 0.08 ^cd^
STJ	3.86 ± 0.08 ^bc^	13.64 ± 0.57 ^e^	5.33 ± 0.50 ^de^	4.44 ± 0.17 ^bc^	7.73 ± 0.87 ^h^	6.54 ± 0.13 ^a^
SM	3.31 ± 0.21 ^cde^	12.27 ± 0.79 ^f^	6.13 ± 0.38 ^c^	5.00 ± 0.40 ^a^	8.54 ± 0.15 ^g^	4.97 ± 0.28 ^e^
WG	3.78 ± 0.18 ^bcd^	14.65 ± 0.71 ^cd^	4.17 ± 0.38 ^g^	4.27 ± 0.17 ^c^	10.38 ± 0.30 ^e^	6.44 ± 0.24 ^ab^
YC	3.28 ± 0.25 ^cde^	15.30 ± 0.50 ^c^	6.03 ± 0.25 ^c^	4.00 ± 0.22 ^cd^	11.74 ± 0.39 ^c^	6.00 ± 0.84 ^cd^

Values are given as mean ± SD (*n* = 3). Different letters in the same column indicate significant differences (*p* < 0.05).

**Table 2:** Color values of sodium alginate/mandarin peel powder films.

Films	L*	a*	b*	Δ*E*
BDZ	70.11 ± 0.25 ^i^	12.51 ± 0.19 ^a^	75.53 ± 0.52 ^d^	83.48 ± 0.38 ^a^
CJ	78.28 ± 0.23 ^b^	3.62 ± 0.45 ^h^	78.35 ± 0.37 ^b^	83.26 ± 0.30 ^a^
DHP	74.06 ± 0.14 ^g^	12.74 ± 0.20 ^a^	77.50 ± 0.05 ^c^	84.27 ± 0.02 ^a^
GG	78.88 ± 0.22 ^a^	0.60 ± 0.42 ^i^	64.99 ± 0.23 ^h^	70.04 ± 0.17 ^b^
JG	74.09 ± 0.02 ^g^	7.51 ± 0.27 ^d^	72.81 ± 0.22 ^e^	79.15 ± 0.24 ^c^
NF	71.61 ± 0.13 ^h^	9.88 ± 0.10 ^c^	77.50 ± 0.08 ^c^	84.57 ± 0.05 ^c^
OG	74.22 ± 0.30 ^g^	4.92 ± 0.06 ^g^	66.05 ± 0.11 ^g^	68.47 ± 0.19 ^b^
PK	77.31 ± 0.31 ^c^	5.93 ± 0.21 ^f^	75.07 ± 0.57 ^d^	80.40 ± 0.64 ^c^
STJ	76.29 ± 0.25 ^e^	6.45 ± 0.17 ^e^	75.17 ± 0.11 ^d^	83.68 ± 0.17 ^c^
SM	74.33 ± 0.02 ^g^	10.70 ± 0.07 ^b^	79.92 ± 0.31 ^a^	86.26 ± 0.30 ^c^
WG	76.75 ± 0.03 ^d^	5.94 ± 0.18 ^f^	68.34 ± 0.71 ^f^	74.03 ± 0.66 ^b^
YC	74.72 ± 0.26 ^f^	6.58 ± 0.47 ^e^	72.70 ± 0.16 ^e^	78.80 ± 0.26 ^c^

Values are given as mean ± SD (*n* = 3 for color values). Different letters in the same column indicate significant differences (*p* < 0.05).

## Data Availability

The data presented in this study are available on request from the corresponding author. The data are not publicly available due to privacy restrictions.

## Supplementary Materials

The following supporting information can be downloaded at: https://www.mdpi.com/article/10.3390/foods13081174/s1, Figure S1: TG (A) and DTG (B) curves of sodium alginate/mandarin peel powder films. Table S1: TGA data for sodium alginate/mandarin peel powder films.
