# Supplementary material for: Preparation, Characterization and Application of Active Food Packaging Films Based on Sodium Alginate and Twelve Varieties of Mandarin Peel Powder
Source: Foods. 2024 Apr 12;13(8):1174. doi: 10.3390/foods13081174 (PMC11048805; doi:10.3390/foods13081174)
Supplement: Supplementary file 1 [file foods-13-01174-s001.zip › foods-2954632-supplementary.pdf]

Supplemental data:

Preparation, characterization and application of active food packaging films based on sodium alginate and twelve varieties of mandarin peel powder

Dawei Yun, Jun Liu *

College of Food Science and Engineering, Yangzhou University, Yangzhou 225127, PR China

* Corresponding author. E-mail: junliu@yzu.edu.cn

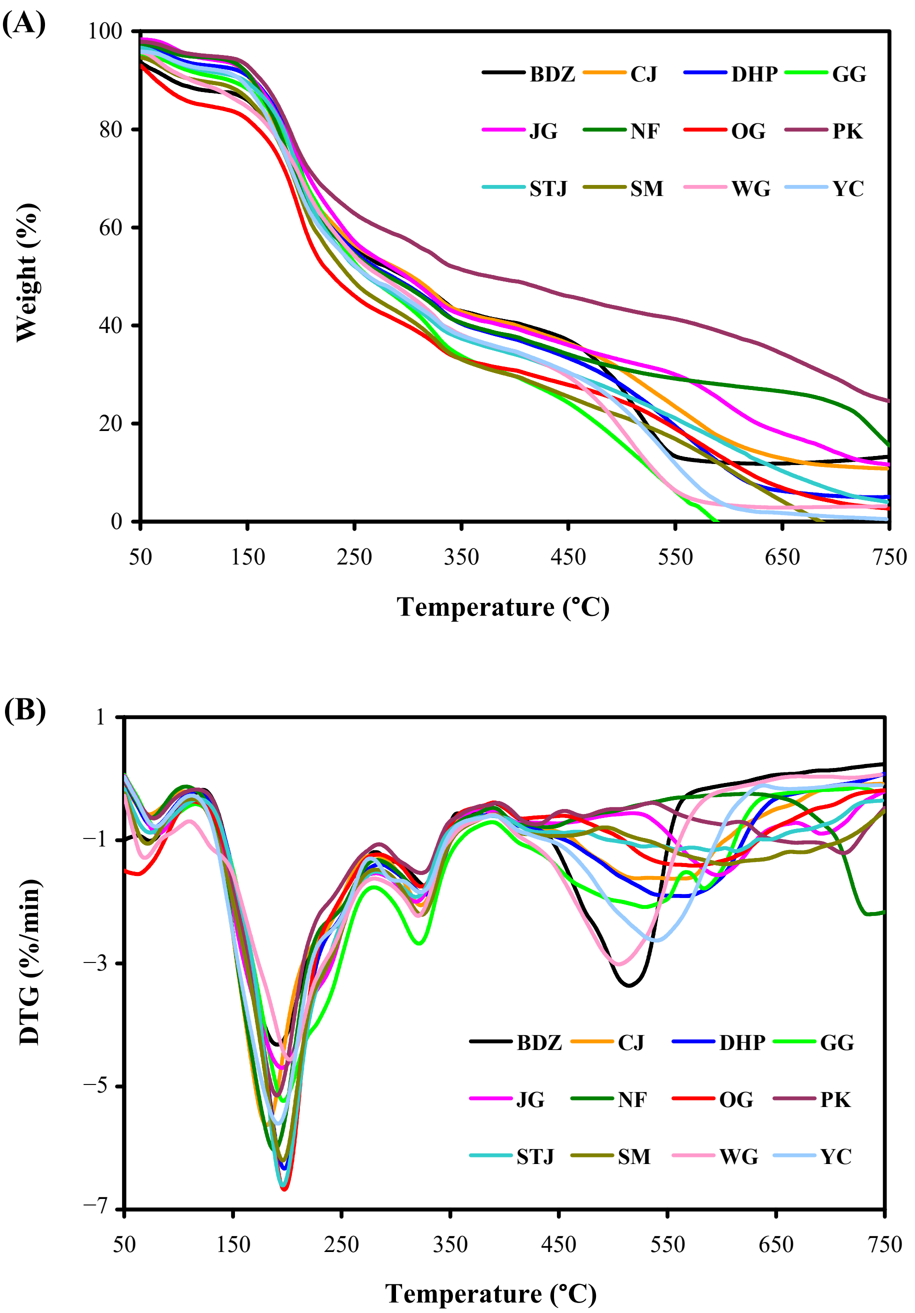

Figure S1. TG (A) and DTG (B) curves of sodium alginate/mandarin peel powder films.

Table S1. TGA data for sodium alginate/mandarin peel powder films.

Film	Stage I		Stage II		Stage III		Stage IV		T_m (°C)	Weight residue at 750 °C (%)
	Range (°C)	Weight loss (%)	Range (°C)	Weight loss (%)	Range (°C)	Weight loss (%)	Range (°C)	Weight loss (%)		
BDZ	50−129	12.41	129−287	36.37	287−395	10.52	395−750	27.44	191	13.26
CJ	50−113	5.32	113−285	42.49	285−387	11.42	387−750	29.95	194	10.82
DHP	50−120	7.12	120−284	42.78	284−392	12.44	392−750	32.58	197	5.08
GG	50−121	9.34	121−286	44.36	286−397	16.45	397−750	29.85	197	0.00
JG	50−123	5.95	123−283	41.94	283−383	11.80	383−750	28.68	195	11.63
NF	50−114	5.35	114−287	45.31	287−396	11.43	396−750	22.39	188	15.52
OG	50−122	15.57	122−288	43.23	288−396	10.23	396−750	28.32	197	2.65
PK	50−123	5.19	123−289	36.31	289−401	9.49	401−750	24.43	190	24.58
STJ	50−125	8.55	125−288	45.14	288−399	12.12	399−750	30.24	196	3.95
SM	50−116	10.46	116−285	46.09	285−397	13.61	397−750	29.84	183	0.00
WG	50−117	11.47	117−288	40.37	288−395	13.20	395−750	31.81	203	3.15
YC	50−116	7.74	116−284	44.67	284−399	12.81	399−750	34.30	192	0.48

T_m: temperature with the maximum decomposition rate.
